# House Cricket (*Acheta domesticus*) as a Promising Functional Ingredient: A Synoptical Review on Nutritional Value, Technological Properties and Sustainability Perspectives

**DOI:** 10.3390/insects17070749

**Published:** 2026-07-22

**Authors:** Filipa Oliveira, Rute F. Vítor, Maria Manuela Silva, Igor Dias

**Affiliations:** 1 Earth Sciences Department of NOVA School of Sciences and Technology, NOVA University Lisbon, Caparica, Campus, 2829-516 Caparica, Portugal; mma.silva@fct.unl.pt; 2 GeoBioTec Research Center, NOVA University Lisbon, 2829-516 Caparica, Portugal; 3 School of Agriculture, Santarém Polytechnic University, Quinta do Galinheiro—S. Pedro, 2001-904 Santarém, Portugal; rute.vitor@esa.ipsantarem.pt (R.F.V.); igor.dias@esa.ipsantarem.pt (I.D.); 4 CIEQV—Life Quality Research Centre, Avenida Dr. Mário Soares n 110, 2040-413 Rio Maior, Portugal; 5 Research Center in Natural Resources, Environment and Society (CERNAS), Santarém Polytechnic University, Quinta do Galinheiro—S. Pedro, 2001-904 Santarém, Portugal

**Keywords:** *Acheta domesticus*, edible insects, novel food, sustainability, cricket powder, protein alternatives

## Abstract

The house cricket (*Acheta domesticus*) is among the edible insect species authorized under the European Union novel food framework and is increasingly explored as a food ingredient. This review synthesizes current evidence on its nutritional composition, techno-functional properties, safety, sustainability, and application potential for human consumption. *A. domesticus* powder is a nutrient-dense ingredient, with reported protein contents ranging from 57.4 to 76.19 g/100 g dry material, together with a relevant amino acid profile, unsaturated lipid fraction, chitin-associated fiber, and selected minerals and vitamins. Its techno-functional profile, including water absorption capacity around 175%, oil absorption capacity around 140%, and emulsifying capacity around 77.7%, supports applications in bakery products, snacks, pasta, meat analogs, and protein-enriched foods. From a sustainability perspective, reported feed conversion ratios around 1.5–1.9 and controlled indoor rearing strengthen its relevance for scalable production, although outcomes depend on substrate choice, energy inputs, production scale, and processing conditions. Safety issues, especially allergenicity, cross-reactivity, microbial control, and contaminant monitoring, remain central to industrial use. Overall, *A. domesticus* powder has emerged and should be regarded as a nutritionally valuable, technologically versatile and promising functional ingredient for sustainable food innovation.

## Simple Summary

The house cricket (*Acheta domesticus*) is an edible insect with great potential for use as a food ingredient. It is already authorized in the European Union in different processed forms and can be incorporated as powder into familiar foods such as bakery products, pasta, snacks, bars and meat-based formulations. This review summarizes current evidence on its nutritional value, technological properties, safety, and sustainability, reinforcing it as a promising functional ingredient. House cricket powder is rich in protein and contains lipids, chitin-associated fiber, minerals, and vitamins. It may also contribute to food formulation through properties such as water and oil absorption and emulsifying capacity. However, its use requires careful control of allergenicity, microbiological safety, lipid oxidation, substrate variability, and production standardization. Overall, *A. domesticus* powder appears to be a promising ingredient for sustainable food innovation, but stronger standardization and more human studies are needed before broader health-related claims can be supported.

## 1. Introduction

The increasing demand for nutritious and sustainable food ingredients has intensified interest in edible insects as alternative sources of protein and other nutrients [1,2,3,4,5,6]. Among them, the house cricket (*Acheta domesticus*) has gained relevance because it is already commercially reared, can be processed into powder, and has been authorized for human consumption under the European Union novel food framework [7,8]. These features make *A. domesticus* one of the best-characterized insect species for food ingredient development.

*A. domesticus* is a hemimetabolous insect belonging to the order Orthoptera [9]. Its biomass is mainly valued for its high protein content, relevant amino acid profile, unsaturated lipid fraction, chitin-associated fiber, and contribution to selected minerals and vitamins [6,10,11,12]. In food applications, cricket powder can be incorporated into familiar products such as bakery products, snacks, pasta, meat analogs, and protein-enriched formulations, where it may improve nutritional density and influence technological performance [13,14,15,16,17,18,19]. It was among the first insect species to receive authorization under EU Novel Food legislation, reflecting its established safety profile [7,8].

Among edible insects, *A. domesticus* has a particularly strong position for food ingredient development because it combines regulatory recognition, commercial rearing, nutritional density, and technological applicability [7,8,9,10,11,12,20,21]. Reported protein contents commonly range from 57.4 to 76.19 g/100 g dry material [11,12,22,23], while its water absorption, oil absorption, and emulsifying capacities support its use in processed food matrices such as bakery products, pasta, snacks, bars, and meat-based formulations [13,14,15,16,17,18,19,20,21,24]. These characteristics make house cricket powder more than a protein alternative; they position it as a versatile ingredient with nutritional and technological relevance.

To better clarify the distinctive position of *A. domesticus* among edible insects, Table 1 summarizes the main features that support its relevance as a food ingredient. The promise of house cricket powder is based on the combination of regulatory recognition, controlled rearing, nutritional value, technological applicability, and compatibility with familiar food products.

This comparative positioning shows that *A. domesticus* is particularly relevant for near-term food innovation because it combines regulatory maturity, controlled production, nutritional density, and practical applicability in processed food matrices.

In this review, the term “functional ingredient” refers to the dual contribution of *A. domesticus* powder to food formulation: techno-functional properties, including water and oil absorption, emulsifying capacity, foaming behavior, and formulation performance [11,13,20,21,24,28,29,30], and emerging bioactive properties, including antioxidant, antihypertensive and gut-related effects [31,32,33,34]. This combined nutritional, technological, and bioactive profile supports the positioning of *A. domesticus* powder as a promising functional ingredient, while health-related effects require stronger validation in human studies.

Although several studies have examined the nutritional composition, safety, sustainability, consumer acceptance, or technological properties of edible insects, the evidence for *A. domesticus* remains dispersed across research areas and rarely integrates how rearing, processing, and formulation conditions jointly shape its nutritional quality, technological performance, and safety profile [21,28,29]. Without this integrative perspective, it is difficult to assess the realistic potential of *A. domesticus* as a food ingredient or to identify the conditions under which its functional properties are reproducible and industrially relevant. This review therefore addresses that gap by providing a focused, critical synthesis across these interconnected dimensions. The added value of this review lies in connecting rearing and processing variables with nutritional composition, techno-functional performance, safety constraints, and realistic industrial applications of *A. domesticus* powder.

Accordingly, this review aims to: (i) synthesize current knowledge on the nutritional composition, techno-functional properties, safety, and sustainability of *A. domesticus* powder; (ii) critically assess the evidence supporting its proposed functional and bioactive effects; (iii) identify the main sources of variability affecting composition and performance; and (iv) highlight current challenges and future research needs for its industrial application as a food ingredient.

## 2. Methodology

This narrative review followed a structured literature search and screening strategy to improve transparency and reproducibility. Peer-reviewed publications were identified in Scopus, Web of Science, and PubMed using combinations of the following keywords: “*Acheta domesticus*”, “house cricket”, “cricket powder”, “cricket flour”, “edible insects”, “nutritional composition”, “amino acids”, “fatty acids”, “minerals”, “vitamins”, “techno-functional properties”, “water absorption”, “oil absorption”, “emulsifying capacity”, “foaming capacity”, “safety”, “allergenicity”, “microbiological quality”, “processing”, “drying”, “life cycle assessment”, “sustainability”, “consumer acceptance”, and “food applications”. The search covered studies published between 2002 and February 2026; 2002 was set as the lower boundary to coincide with the publication of the foundational definition of functional foods adopted in this review, ensuring conceptual consistency in the interpretation of evidence across the included studies. Backward and forward citation tracking of key articles and recent reviews was also performed. European Union legal texts and EFSA scientific opinions relevant to *A. domesticus* were retrieved from official sources and included regardless of publication year.

The initial search yielded over 300 records; after removal of duplicates and screening of titles, abstracts, and full texts, approximately 97 publications and official sources were retained and used to support this review. Studies were included when they reported compositional, processing, techno-functional, safety, sustainability, consumer-related or food-application data for *A. domesticus* intended for human consumption, including whole insects, powders, flours, defatted powders, and protein fractions. Studies on other cricket species or edible insects were not used as primary evidence for *A. domesticus* but were included when they provided direct comparative data useful to contextualize its nutritional, technological, safety or sustainability profile. Studies focused exclusively on animal feed, non-food uses, or insect species without relevance for comparison with *A. domesticus* were excluded.

Because studies differed in analytical methods, processing conditions, ingredient format, and reporting units, the data were synthesized qualitatively as indicative ranges, not as directly comparable fixed values. Attention was given to unit harmonization in nutritional tables and to identifying cases where dry basis, wet basis, or “as received” values were not clearly distinguished. Unit inconsistencies or comparability problems were flagged in the text or table notes.

The retained literature was organized according to the main themes of the review: regulation, biology and production, nutritional composition, safety, techno-functional properties, sustainability, food applications, consumer acceptance, and future research needs. This structure allowed the evidence to be assessed from an application-oriented perspective, with emphasis on how rearing, processing and formulation conditions influence the industrial potential and limitations of *A. domesticus* as a food ingredient.

## 3. Regulations

In the European Union, edible insects intended for human consumption are regulated under the Novel Food Regulation (EU) 2015/2283, which requires pre-market authorization for foods not consumed to a significant degree in the EU before 15 May 1997 [30]. Insect-based foods must additionally comply with general food hygiene requirements, including HACCP-based procedures under Regulation (EC) No 852/2004, and with food information and labeling rules under Regulation (EU) No 1169/2011 [31,32].

For *Acheta domesticus*, two product-specific authorizations are currently in force. Commission Implementing Regulation (EU) 2022/188 authorizes the placing on the market of frozen, dried, and powder forms of the house cricket as a novel food, defining permitted food categories, maximum use levels, compositional specifications, and labeling requirements [7]. Commission Implementing Regulation (EU) 2023/5 extends this framework to partially defatted *A. domesticus* powder, a distinct ingredient form with different compositional and functional characteristics [8]. Both regulations require that allergen information be communicated on the label, given the documented cross-reactivity between cricket proteins and those of crustaceans, mollusks, and dust mites, which constitutes a legal obligation with direct implications for product design, ingredient declaration, and consumer communication [7,8,22].

These authorizations establish market access but simultaneously impose production and processing requirements that are not trivial to meet at industrial scale. Compliance requires substrate traceability, hygienic rearing conditions, validated inactivation and drying protocols, contaminant monitoring, and consistent allergen quantification and labeling [7,8,31,33,34,35]. In practice, many small and medium producers lack the standardized analytical infrastructure to demonstrate compliance across all these dimensions simultaneously, which limits market entry despite formal authorization [34,36].

Outside Europe, regulatory approaches differ substantially. A relevant distinction exists between unprocessed insects, which in some regions are traded under agricultural or traditional food frameworks without specific food safety authorization, and processed insect-based products intended for human consumption, which typically fall under food law and may require safety assessment, specific authorization, or labeling [36,37,38]. Conflating these two categories risks misrepresenting the actual regulatory burden at different stages of the supply chain. Country-specific frameworks range from dedicated insect farming and food standards, such as Thailand’s TAS 8202-2017 for cricket farms, to permitted species lists and import conditions, as in the Singapore Food Agency framework [37,39]. In many markets, insect-based foods remain governed by general food law without insect-specific provisions, creating regulatory uncertainty that affects trade and investment [36,38].

Overall, *A. domesticus* has a more advanced regulatory status in the European Union than most other edible insect species, with two product-specific authorizations already in force [7,8]. These authorizations provide a strong basis for market development, while further harmonization of analytical methods for proximate composition, allergen quantification, and contaminant monitoring would strengthen industrial standardization. Alignment of labeling practices across Member States is also important for consistent product quality and consumer confidence in cricket-based ingredients [40,41,42]. The main EU regulatory milestones relevant to the commercialization of *A. domesticus*-derived food ingredients are summarized in Table 2.

## 4. Biology, Nutritional Profile, Technological Properties and Bioactive Potential

### 4.1. Biology and Production Considerations

The biological characteristics of *Acheta domesticus* and the conditions used during production directly affect the quality of the final ingredient. Life cycle stage, rearing temperature, humidity, density, substrate composition, killing method, and drying process influence biomass yield, microbial safety, nutrient composition, and techno-functional performance. These factors are integral to ingredient standardization, not merely production background.

#### 4.1.1. Life Cycle

*Acheta domesticus* is a hemimetabolous insect that develops through egg, nymphal, and adult stages, without a pupal phase [9]. Under controlled conditions, development from hatch to adult generally involves 8–9 nymphal instars and can take approximately 45 days at around 30 °C. When egg incubation is included, the full egg-to-adult cycle is usually close to 57–60 days [40].

The developmental stage at harvest is relevant for ingredient quality. Younger and adult stages may differ in body composition, particularly in protein, lipid, and chitin fractions. This affects powder composition, digestibility, texture, and technological behavior. For industrial production, harvesting at a defined stage is therefore important to improve batch homogeneity and reduce variability between production cycles [41,42].

#### 4.1.2. Farming Conditions and Management

*A. domesticus* is usually reared indoors in boxes, containers or cages, which allows better control of feeding, ventilation, hygiene, and pest management [9]. Practical production conditions commonly target approximately 30 ± 2 °C and 50–60% relative humidity, although egg hatching and early development may require higher humidity levels [43,44].

Temperature, humidity, and density affect growth rate, survival, and feed conversion in *A. domesticus* [25,43,44,45]. Suboptimal conditions can prolong development, increase mortality, and reduce biomass yield, while inadequate humidity control may compromise both development and hygienic quality [25,43,44]. Therefore, environmental control is directly linked to productivity, microbiological quality, and consistency of the final cricket powder [25,45].

Population density is another critical parameter. Higher densities may improve space efficiency, but they can also increase stress, cannibalism, disease transmission, and variability in individual growth [45,46]. For this reason, density should be optimized according to developmental stage and production objective, rather than maximized without control [45,46].

#### 4.1.3. Substrate Selection and Regulatory Constraints

Substrate composition is one of the main determinants of production efficiency and nutritional composition in *A. domesticus*. Diet influences growth, feed conversion, survival, and the final lipid, mineral, and protein profiles of the biomass [23,26,47,48]. Therefore, differences in feed composition partly explain the variability reported between nutritional studies [11,12,22,23,48].

In the European Union, substrates used for edible insect production must comply with feed hygiene and animal by-product rules, since farmed insects are considered farmed animals [49,50,51]. This restricts the use of some waste streams, including manure and catering waste, and reduces the practical use of highly circular substrates [49,50,51]. Agro-industrial side streams may be relevant, but they require risk-based selection, traceability, and monitoring for biological and chemical contaminants [1,34,52].

From an industrial perspective, substrate choice involves a trade-off between nutritional consistency, safety, and sustainability. Standardized feeds may improve growth performance and compositional reproducibility, but they can reduce sustainability advantages if they compete with conventional feed or food resources [1,34,52,53]. Conversely, low-cost side streams may improve circularity, but can increase variability and safety-monitoring needs [34,49,50,51,52]. This makes substrate standardization central to both sustainability claims and ingredient quality [34,52,53].

#### 4.1.4. Killing and Primary Processing

Killing and primary processing are critical control points in the production of *A. domesticus* powder because they influence microbial reduction, enzyme activity, browning reactions, nutrient retention, lipid oxidation, and protein functionality [27,52,54,55,56,57,58]. Therefore, the processing method selected affects not only safety, but also the nutritional and technological quality of the final ingredient [27,52,56,57].

Blanching at 100 °C for short periods has been reported to reduce yeasts and molds, limit browning, and improve chitin purity, although losses of minerals and free amino acids may occur [52]. Freezing at −20 °C can preserve several nutrients but may be less effective as a microbial reduction step and may contribute to browning or amino acid degradation during subsequent processing [54,55]. Freeze-drying generally preserves protein structure and thermolabile compounds better, but its high energy demand may limit industrial feasibility [56,57].

Drying conditions also affect ingredient quality. Hot-air, vacuum, microwave, and freeze-drying can lead to different protein contents, antioxidant activity, color, and technological properties in cricket powder [27]. In addition, oven-dried powders may show higher peroxide values and aldehyde formation than lyophilized powders, while lower storage temperatures can reduce oxidative deterioration [57,58]. Thus, industrial protocols should balance safety, nutrient retention, functional performance, shelf-life, and energy cost [27,56,57,58].

In summary, no single killing or drying method simultaneously optimizes microbiological safety, nutrient retention, lipid stability, and techno-functional performance. Freeze-drying best preserves protein structure and functional properties but is energy-intensive, while oven-drying and tray-drying offer cost-effective alternatives with comparable nutritional outcomes [27,49,52]. These trade-offs should be explicitly considered when developing industrial protocols or comparing data across studies.

#### 4.1.5. Feed Efficiency, Genetics, and Health

Feed efficiency is one of the main arguments supporting the production of *A. domesticus* as a food ingredient. Reported feed conversion ratios (FCR) are commonly around 1.5–1.9 and vary with diet composition, temperature, humidity, density, developmental stage, and production management [25,26,27]. Evaluating FCR together with substrate composition, biomass yield, and nutrient profile provides a more meaningful measure of production efficiency.

Diet quality strongly affects production performance. Diets with adequate protein levels may reduce development time, increase body weight, and improve feed conversion, whereas low-protein or poorly balanced diets may increase FCR and reduce survival [23,26]. These interactions reinforce the importance of substrate standardization not only for nutritional consistency, but also for productive efficiency.

Genetic variability is another relevant factor for industrial standardization. Commercial colonies may differ in growth rate, body size, survival, reproductive performance, and nutrient composition, which can affect both productivity and batch consistency [29,59,60]. The availability of genomic resources and selective breeding tools for *A. domesticus* may support future selection strategies aimed at improving productive robustness, disease resistance and nutritional uniformity [29,59,60]. Selection programs should preserve genetic diversity to avoid inbreeding and reduced colony resilience [29,59,60].

Animal health remains a major constraint for scale-up. *Acheta domesticus* densovirus (AdDV) is a relevant pathogen in commercial cricket production and has been associated with mortality outbreaks and production losses [61,62]. Rearing density and environmental conditions can influence virus prevalence, while molecular tools such as qPCR may support early detection and biosecurity monitoring [62,63]. For industrial production, routine health surveillance, controlled density, and traceable colony management are therefore essential to reduce losses and improve ingredient consistency [46,61,62].

Overall, feed efficiency, genetics, and colony health are interconnected determinants of scalability. Improving *A. domesticus* production requires standardized rearing protocols, disease monitoring, and selection strategies that support stable biomass yield and reproducible ingredient quality.

### 4.2. Nutritional Values

*Acheta domesticus* powder is generally described as a nutrient-dense ingredient, mainly because of its high protein content, relevant lipid fraction, chitin-associated fiber, and contribution to selected minerals and vitamins [10,11,12,22]. Reported proximate values show that protein commonly represents the major macronutrient fraction, often above 57% of dry matter, while fat, ash, carbohydrates, and fiber show wider variation between studies [11,12,22,23]. This nutritional profile supports the use of house cricket powder in protein-enriched foods, with composition best reported as a defined range rather than a single fixed value.

Several factors explain this variability. Nutritional composition may differ according to breeding method, sex, developmental stage, and diet [42]. Differences between egg, nymphal, and adult stages may also influence nutrient composition, which is relevant when defining the harvest stage for food ingredient production [41]. Diet is particularly important because *A. domesticus* can convert supplied nutrients into different body composition profiles [47]. For example, changes in protein formulation may affect body weight and production yield, even when final lipid and mineral contents remain relatively stable [26].

Environmental conditions also influence nutritional outcomes indirectly through their effects on growth, survival, and biomass yield. Temperature and rearing density affect developmental performance and metabolic responses [25], while elevated temperatures and density can also influence survival, growth, and the prevalence of *Acheta domesticus* densovirus [45]. Therefore, diet, temperature, density and colony health should be considered together when interpreting differences in body composition, including lipid and ash content [23,25,45,48].

Before comparing nutritional data across studies, the analytical basis must be clearly defined. Values reported on a dry matter basis, wet matter basis, or “as received” basis are not directly interchangeable, especially for proximate composition, because moisture affects the apparent concentration of protein, fat, ash, fiber, and carbohydrates [11,12,22,23]. Accordingly, the values presented in the following tables are used as indicative ranges, not as directly comparable fixed standards.

#### 4.2.1. Macronutrients

The proximate composition of *A. domesticus* powder reported in different studies is summarized in Table 3. Values are expressed as g/100 g dry material, except when otherwise indicated by the original source.

The data presented in Table 3 show that protein is the most abundant macronutrient in *A. domesticus* powder, ranging from 57.4 to 76.19 g/100 g dry material across the selected studies [11,12,22,23]. This confirms the relevance of *A. domesticus* powder as a protein-rich ingredient for food formulation. The observed range also reflects the influence of production system, diet, processing conditions, and analytical reporting [11,12,22,23,26].

Fat content shows greater variability, ranging from 8.9 to 31.28 g/100 g dry material [11,12,22,23]. This variation is important from an industrial perspective because higher lipid levels can increase energy density and influence texture, while also increasing susceptibility to lipid oxidation during processing and storage [26,64,65]. Lipid content should therefore be evaluated together with drying conditions, packaging, and expected shelf-life.

Carbohydrate and fiber values are also variable. In Table 3, fiber ranges from 3.7 to 16.4 g/100 g dry material, while carbohydrates range from 1.6 to 19.1 g/100 g dry material [11,12,22,23]. The higher carbohydrate and fiber values reported by Andrade et al. [12] may be related to the specific diets used, differences in substrate composition or analytical calculation methods. Since chitin contributes to the fiber fraction, differences in developmental stage, processing, and analytical method may also influence the apparent fiber content [10,57,58,63,64,66].

Overall, the macronutrient profile of *A. domesticus* powder supports its use in protein enrichment and food product development. The variability observed in fat, carbohydrates, and fiber highlights the need to define cricket powder by diet, harvest stage, processing method, moisture content, and analytical basis to improve comparability and ingredient standardization [11,12,22,23].

#### 4.2.2. Minerals

*A. domesticus* powder contains nutritionally relevant minerals, including phosphorus, potassium, magnesium, calcium, iron, and zinc [10,11,12]. As shown in Table 4, phosphorus is consistently one of the most abundant minerals, while zinc and iron are present at levels that may contribute to the nutritional value of cricket-based ingredients [10,11,12].

Mineral variability is particularly important for food formulation and labeling. Calcium, potassium, magnesium, and sodium show marked differences between the studies summarized in Table 4, whereas iron and zinc appear less variable across the selected datasets [10,11,12].

The data in Table 4 indicate that *A. domesticus* powder may be a relevant source of phosphorus, potassium, magnesium, iron, and zinc [10,11,12]. The magnitude of variation differs between minerals: potassium ranges from 126.62 to 1145.28 mg/100 g dry material, while calcium ranges from 149.75 to 238.25 mg/100 g dry material [10,11,12]. This suggests that some minerals are more sensitive to production conditions and feed composition than others.

Dietary strategies such as gut loading may be used to modify the mineral profile of crickets, particularly for minerals such as calcium and phosphorus [11,35]. This approach can support the development of ingredients with targeted nutritional profiles. Mineral enrichment strategies require control because they can also affect growth performance, mineral balance, safety monitoring, and product standardization [11,23].

From an industrial perspective, mineral composition has direct implications for nutrition claims, labeling, and quality control. Producers should therefore report feed composition, harvest stage, processing conditions, and analytical methods together with mineral data [66,67,68,69]. This would improve comparability between studies and support more reliable use of *A. domesticus* powder in mineral-enriched food formulations [10,11,12].

#### 4.2.3. Vitamins

*A. domesticus* has been reported to contain several vitamins, particularly B-group vitamins, vitamin E and choline [10,66,67]. Vitamin data extend the nutritional profile of house cricket powder, although available studies differ in sample condition, analytical methods and reporting format [10,66,67]. Vitamin composition is therefore best presented as evidence of nutritional potential rather than as a fixed compositional standard.

Table 5 summarizes the vitamin composition reported for *A. domesticus*. The table includes data from Payne et al. [67], Ververis et al. [68], and Magara et al. [10], while distinguishing between undried and dried matter when reported by the original source.

The data presented in Table 5 indicate that *A. domesticus* may contribute to the intake of several vitamins, particularly B-group vitamins, vitamin E, and choline [10,67,68]. Among the B-group vitamins, riboflavin, niacin, and pantothenic acid are the most consistently reported across sources, although the magnitude of the values varies between studies and sample conditions [10,67,68].

The comparison between undried and dried matter reported by Ververis et al. [68] shows that sample condition can strongly influence apparent vitamin concentration. This is particularly relevant for vitamins reported across wide ranges, such as vitamins B2, B12, and E, and reinforces the need to clearly indicate the moisture basis when presenting vitamin data [68].

Values preceded by “<“ represent limits reported in the original source and should not be treated as quantified concentrations. In addition, the high variability observed for some vitamins shows that current data are not sufficient to define a stable vitamin profile for *A. domesticus* powder [10,67,68].

From an industrial and nutritional perspective, vitamin data are most useful as evidence of nutritional potential rather than fixed compositional standards. Future studies should report analytical methods, sample basis, detection limits, and units more consistently, especially when vitamin composition is used for nutritional labeling or product positioning [10,67,68].

### 4.3. Bioactive Potential

The bioactive profile of *A. domesticus* is supported primarily by preclinical evidence. Cricket proteins and derived hydrolysates may release peptides with antioxidant, antihypertensive or gut-related effects [31,32,33]. Most available data come from in vitro assays, cell models or animal studies, with fewer human intervention studies [31,32,33,34,68]. *A. domesticus* is therefore best positioned as a nutrient-dense ingredient with emerging bioactive potential.

#### Bioactive Potential (Preclinical Evidence)

Protein hydrolysates obtained from *A. domesticus* have shown in vitro angiotensin-converting enzyme (ACE)-inhibitory activity and antioxidant-related effects, including the identification of short peptides with predicted biological activity [70]. In animal models, *A. domesticus* protein hydrolysates have been associated with reduced blood pressure, improved vascular function, and lower oxidative-stress markers [71]. These findings suggest potential physiological relevance, but they do not establish direct health effects in humans.

Evidence related to gut health is also emerging. In controlled human intervention, cricket consumption increased *Bifidobacterium animalis* abundance and reduced circulating TNF-α, suggesting possible effects on gut microbiota and inflammation-related markers [31]. The available human evidence remains early, and the effects can depend on dose, matrix, processing, chitin content, and individual microbiota composition. Stronger evidence from well-designed human trials is needed to support health-related claims [31,34,72].

Phenolic compounds have also been detected in *A. domesticus*, including phenolic acids and flavonoids such as p-coumaric acid, ferulic acid, chlorogenic acid, quercetin, naringenin, and apigenin [73]. Because these compounds are reported at low concentrations and probably reflect dietary carry-over from plant-based substrates, they are better interpreted as minor contributors to the bioactive profile of cricket powder rather than primary markers of its functionality [73,74].

Overall, current evidence supports *A. domesticus* as a source of protein-derived and gut-related bioactive potential. Antioxidant, antihypertensive, and gut-related effects are promising, with future studies needed to evaluate bioaccessibility, peptide stability during digestion, dose–response effects, food matrix interactions, and reproducibility in human populations [32,33,34,72].

### 4.4. Techno-Functional Properties, Quality and Food Applications

Beyond nutritional composition, the industrial value of *A. domesticus* powder depends on its techno-functional behavior, physicochemical stability, microbiological quality, and performance in food matrices [13,75,76,77]. These properties determine whether cricket powder can be used not only as a protein-rich ingredient, but also as a functional component affecting hydration, texture, emulsion stability, shelf-life, and sensory quality.

Key techno-functional properties include water absorption capacity, oil absorption capacity, protein solubility, emulsifying capacity, foaming capacity, and foam stability [11,13,20,21,24,72,75,76]. These parameters are relevant because they influence dough hydration, mouthfeel, fat retention, aeration, and structural stability in products such as bakery goods, snacks, meat analogs, and hybrid formulations [13,21,24,72,75,76].

Reported values vary across studies because techno-functional properties depend on cricket composition, particle size, protein structure, lipid content, drying method, defatting, and analytical protocol [47,69,77,78,79,80]. The values summarized in Table 6 are therefore used as indicative benchmarks for formulation, not as fixed standards.

Physicochemical and microbiological quality are also central to industrial application. Low moisture and low water activity support shelf stability, while microbial counts, pathogen absence, and spore-forming bacteria determine safety and storage suitability [24,57,82]. In addition, lipid oxidation is a major quality limitation because *A. domesticus* contains unsaturated fatty acids that may generate off-flavors and reduce sensory acceptance during storage [51,52,69,73].

The data summarized in Table 6 show that *A. domesticus* powder has relevant water and oil absorption capacities, which support its use in products where hydration, mouthfeel, and fat retention are important [20,21]. Its emulsifying capacity also supports applications in systems containing both aqueous and lipid phases, such as meat emulsions, sauces, or hybrid products [20,21,82]. Foaming capacity is lower than its hydration and emulsifying performance, indicating that cricket powder can contribute to aerated systems, but requires formulation adjustments when replacing highly functional foaming proteins [20,21].

Protein solubility is a key determinant of emulsifying and foaming behavior. Reported solubility values are moderate and strongly influenced by pH, with lower solubility near the isoelectric region and improved solubility under alkaline conditions [11,21,81]. This means that the functionality of cricket powder is matrix-dependent and may vary substantially between acidic, neutral, and alkaline food systems.

From a quality perspective, low moisture and water activity support shelf stability, but they do not eliminate the need for microbial and oxidative control [20,21,57,82]. Lipid oxidation remains one of the main limitations for cricket-derived ingredients, particularly during drying, milling, and storage [57,58,69,83]. Packaging strategies that reduce oxygen exposure, together with appropriate storage temperature and oxidation monitoring, are therefore important for maintaining nutritional and sensory quality [57,58,69,83].

Taken together, these data support the use of *A. domesticus* powder as a technologically useful ingredient, with performance governed by processing, composition, formulation conditions, and the specific technological objective of the final product [13,21,24,28,29,30].

#### 4.4.1. Microbiological Quality and Storage Stability

The microbial load of live or raw *A. domesticus* can be high, with total aerobic counts and *Enterobacteriaceae* commonly reported around 10^7^–10^8^ CFU/g, while yeasts and molds may also be present [78,79]. These values underline the importance of controlled processing before raw cricket biomass is used as a food ingredient.

Thermal treatments such as steaming, blanching and drying markedly reduce total viable counts and *Enterobacteriaceae*, while the absence of major foodborne pathogens depends on hygienic rearing, substrate quality, validated processing, and prevention of post-processing contamination [73,78,79,81]. Storage stability is further supported by low residual moisture, low water activity, suitable packaging, and routine microbial testing, including monitoring of spore-forming bacteria such as *Bacillus cereus* [64,84,85,86,87,88].

#### 4.4.2. Food Applications

*A. domesticus* powder has been incorporated into several food matrices, mainly with the aim of increasing protein content, partially replacing conventional ingredients or improving specific technological properties [13,14,15,16,17,18,19]. The available studies include bakery products, pasta, meat emulsions, beef patties, shortbread cookies, gluten-free bars, and chocolate chip cookies (Table 7). These applications show that cricket powder is more suitable as an incorporated ingredient than as a visible whole-insect product, which is also relevant for consumer acceptance [14,16,17,18,19,85].

The technological and sensory impact of cricket powder depends strongly on the incorporation level and food matrix. Moderate inclusion levels can improve nutritional density while maintaining acceptable product quality, but higher levels may darken color, modify flavor, change texture, or reduce acceptability [13,14,16,17,18,19]. Therefore, formulation studies should define not only the percentage of incorporation, but also the technological objective, such as protein enrichment, water retention, texture modification, or partial replacement of meat, fat, or cereal flour.

The data summarized in Table 7 indicate that *A. domesticus* powder can be used in both cereal-based and meat-based formulations [13,14,15,16,17,18,19]. In bakery and cereal products, cricket powder generally increases protein content, while higher incorporation levels can reduce dough extensibility, increase firmness, and darken the final product [13,14,16]. These effects are relevant because color, flavor, and texture are key determinants of consumer acceptance.

In meat-based products, cricket powder can contribute to water and oil retention, texture modification, and partial replacement of meat or fat [15,17]. These properties are consistent with the water and oil absorption capacities described for cricket powder. Formulation optimization helps prevent undesirable changes in color, flavor, pH, or firmness [15,17].

Overall, the most promising applications are familiar processed foods in which cricket powder is not visually identifiable, such as bakery products, snacks, bars, pasta, meat analogs, and hybrid products [13,14,15,16,17,18,19,21]. Successful incorporation depends on controlling particle size, lipid oxidation, flavor, color, and inclusion level. Future product-development studies should combine nutritional enrichment with sensory analysis, shelf-life testing, and consumer acceptance evaluation [14,16,17,18,19,21].

## 5. Sustainable Utilization of *Acheta domesticus*: Safety, Scalability and Consumer Acceptance

Functional foods are generally defined as foods that may provide benefits beyond basic nutrition when consumed as part of the usual diet [86]. In the case of *Acheta domesticus*, this concept should be applied with caution. Current evidence supports house cricket powder primarily as a nutrient-dense and technologically versatile ingredient, rather than as a clinically validated functional food. Therefore, its functionality should be understood as the combined result of nutritional value, techno-functional performance, emerging bioactive potential, safety control, processing conditions, and suitability for incorporation into realistic food matrices.

The nutritional relevance of *A. domesticus* powder is supported by its high protein content, relevant amino acid profile, unsaturated lipid fraction, chitin-associated fiber, and contribution to selected minerals and vitamins [10,11,12,24,75]. These characteristics justify their use in protein-enriched formulations and in products where nutritional improvement is a major objective. However, the nutritional functionality of cricket powder cannot be interpreted as a fixed species trait, because composition varies with diet, developmental stage, rearing conditions, substrate, killing method, drying process, and ingredient format [21,24,48,49,70,87]. For this reason, *A. domesticus* powder should be considered a family of ingredient formats rather than a single standardized ingredient category.

Its techno-functional value is also central to its positioning as a functional ingredient. Water absorption capacity, oil absorption capacity, protein solubility, emulsifying capacity, foaming behavior, and formulation performance support its use in bakery products, snacks, pasta, meat analogs, hybrid products, and protein-enriched foods [11,13,20,28,29,71,72,75]. These properties are relevant because they influence dough hydration, mouthfeel, fat retention, emulsion stability, aeration, texture, and structural stability in food matrices. Nevertheless, this functionality is application-dependent and varies with powder composition, particle size, lipid content, protein structure, drying method, defatting, analytical protocol, and the characteristics of the food matrix [13,29,62,63,66,75]. Thus, the functional performance of cricket powder must be evaluated in relation to specific formulations rather than assumed from compositional data alone.

Potential bioactive effects provide an additional, but less established, dimension of functionality. Protein hydrolysates from *A. domesticus* have shown antioxidant and angiotensin-converting enzyme inhibitory activity in vitro, and animal studies suggest possible antihypertensive and oxidative-stress-related effects [31,32]. Evidence related to gut health is also emerging, including a controlled human intervention reporting changes in gut microbiota and inflammatory markers after cricket consumption [30]. Phenolic compounds have also been detected in *A. domesticus*, although they are probably present at low concentrations and may reflect dietary carry-over from plant-based substrates rather than being primary markers of cricket functionality [89,90]. Overall, antioxidant, antihypertensive, and gut-related effects are promising, but the available evidence remains mainly preclinical or based on limited human studies [22,30,31,32,84]. Health-related claims should therefore remain cautious until supported by stronger human intervention trials.

Safety is a prerequisite for translating this functional potential into food applications. EFSA assessments concluded that authorized *A. domesticus* products are safe under the proposed conditions of use, but allergenicity remains a key concern [63,73,76]. Cricket proteins may cause primary sensitization and may cross-react with allergens from crustaceans, mollusks and dust mites; therefore, EU authorizations require specific allergen information on the label [7,8,73]. Microbiological safety also depends on hygienic rearing, substrate quality, validated killing, and drying steps, adequate storage and prevention of post-processing contamination [57,78,79,91,92]. Chemical safety is linked to feed and rearing conditions, since heavy metals or other contaminants may originate from substrates or the production environment [81,91]. Consequently, the safe use of *A. domesticus* as a functional ingredient requires integrated control of allergens, microbial hazards, chemical contaminants and processing conditions throughout the production chain [40,57,73,82].

Scalability and sustainability further determine whether the functional potential of *A. domesticus* can be reproduced at industrial level. Although house cricket is frequently presented as an efficient and sustainable protein source, industrial scalability depends on more than feed conversion efficiency. Production costs and environmental performance are influenced by substrate choice, climate control, labor, energy use, drying method, processing efficiency, and final product yield [33,45,54,69,83,88,93]. Standardized feeds may improve growth, survival, and compositional consistency, but may reduce sustainability advantages if they compete with conventional feed or food resources [54,59,88]. Conversely, agro-industrial side streams can improve circularity, but they require traceability and contaminant monitoring [38,50,51,52,53]. Therefore, sustainability should not be treated separately from functionality, because rearing and processing conditions directly affect ingredient composition, safety and techno-functional performance.

Consumer acceptance is the final step in translating functional potential into marketable products. Acceptance is generally higher when insects are incorporated into familiar foods in non-visible forms, such as powders, bars, bakery products, pasta or protein-enriched formulations, rather than presented as whole insects [75,94,95,96]. This makes *A. domesticus* powder especially relevant for product development, as it allows nutritional enrichment while reducing visual rejection. Studies using cricket powder in bread, pasta, muffins, biscuits, cookies, bars, and meat products show that moderate incorporation levels can improve nutritional value while maintaining acceptable technological and sensory properties [13,14,15,16,17,18]. However, higher inclusion levels may darken color, intensify characteristic flavor, modify texture, and reduce consumer liking [13,14,16,17,18,19,72]. Successful commercialization will therefore depend on sensory optimization, appropriate incorporation levels, control of lipid oxidation, clear allergen communication, transparent sustainability claims, and consumer education [75,85,86,94,95,96,97,98].

Taking together, the available evidence supports the positioning of *A. domesticus* powder as a promising functional ingredient, but not yet as a clinically validated functional food. Its strongest current functionality lies in the convergence of nutritional density and techno-functional performance, while bioactive effects remain promising but insufficiently confirmed in humans. The main challenge is therefore not only to demonstrate potential health-related benefits, but also to standardize rearing, processing, formulation, and safety control so that nutritional, technological, and bioactive properties become reproducible under realistic industrial and consumption conditions.

## 6. Conclusions

*Acheta domesticus* is a promising functional food ingredient, as it combines nutritional density, techno-functional performance, regulatory recognition and practical applicability in familiar food matrices. Recent evidence shows that house cricket powder is a protein-rich ingredient, with reported protein contents ranging from 57.4 to 76.19 g/100 g dry material, together with relevant lipid, chitin-associated fiber, mineral and vitamin fractions. Its water absorption, oil absorption and emulsifying capacities further support its use in bakery products, snacks, pasta, meat analogs, and protein-enriched formulations.

Compared with many edible insects that remain less characterized or mainly associated with traditional consumption, *A. domesticus* has a more realistic pathway for near-term food innovation. Its authorization under the European Union novel food framework, compatibility with controlled indoor farming, and suitability for powder-based applications strengthen its industrial relevance. These features support its positioning not only as an alternative protein source, but also as a versatile ingredient platform for sustainable food development.

The main challenge is no longer whether *A. domesticus* has potential, but how to standardize and control this potential across production and processing systems. Diet, developmental stage, rearing conditions, substrate, killing method, drying process, and ingredient format influence composition, safety and technological behavior. Future progress should therefore focus on harmonized analytical methods, standardized rearing and processing protocols, allergen and microbial control, oxidation management, sensory optimization, and stronger human evidence for proposed bioactive effects. Overall, *A. domesticus* represents one of the most promising edible insect ingredients for functional and sustainable food innovation.

## Figures and Tables

**Table 1:** Comparative positioning of *Acheta domesticus* among edible insects for food ingredient development.

Dimension	Distinctive Position of *A. domesticus*	Relevance for Food Ingredient Development
Regulatory and market readiness	*A. domesticus* is authorized in the European Union in frozen, dried and powder forms, as well as partially defatted powder [7,8].	Provides a clearer regulatory basis for food ingredient development than many less characterized edible insects.
Production characteristics	*A. domesticus* can be reared indoors under controlled conditions and show favorable feed conversion efficiency, although performance depends on diet and management [9,25,26,27].	Supports scalable production, traceability and batch standardization.
Nutritional and technological profile	*A. domesticus* powder is protein-rich and shows useful water absorption, oil absorption and emulsifying properties [10,11,12,20,21,22].	Supports its use as a nutrient-dense and techno-functional ingredient.
Food applications and acceptance	Cricket powder has been incorporated into familiar foods such as bread, pasta, muffins, biscuits, cookies, bars and meat products [13,14,15,16,17,18,19,24].	Non-visible incorporation into familiar matrices may improve consumer acceptance compared with whole-insect consumption.

**Table 2:** Key EU regulatory milestones for the commercialization of *Acheta domesticus*-derived food ingredients.

Regulation (EU)	Scope and Main Provisions	Relevance for *A. domesticus* Ingredients
1169/2011	Food information to consumers	Defines general labeling requirements, including relevant allergen information.
852/2004	Hygiene of foodstuffs	Establishes hygiene and HACCP-based requirements for food operators.
2015/2283	General Novel Food Regulation	Requires pre-market authorization for novel foods and EFSA safety assessment.
2022/188	Specific authorization	Authorizes frozen, dried and powder forms of *Acheta domesticus* as a novel food.
2023/5	Expansion of authorization	Authorizes partially defatted *Acheta domesticus* powder as a novel food.

**Table 3:** Proximate composition of *A. domesticus* powder reported in selected studies.

Macronutrients (g/100 g Dry Material)	Udomsil et al. [11]	Bawa et al. [23]	Turck et al. [22]	Andrade et al. [12]	Andrade et al. [12]
Moisture	6.3	---	4.2	5.0	4.3
Ash	5.4	2.9–4.6	3.9	4.4	4.8
Crude Protein	71.7	70.38–76.19	60.26	57.4	60.8
Fat	10.4	8.9–19.3	31.28	14.1	13.4
Carbohydrates	1.6	5.2–10.2	2.1	19.1	16.8
Fiber	4.6	3.7–7.5	4.6	14.8	16.4

Note: The five breeding systems included variations in feeding regime, stocking density, and rearing conditions, allowing the assessment of how these factors influence the proximate composition of *Acheta domesticus*. The last two columns are from the same author but refer to different diets.

**Table 4:** Minerals composition of *Acheta domesticus* in four different breeding systems.

Elementals (mg/100 g Dry Material)	Udomsil et al. [11]	Magara et al. [10]	Andrade et al. [12]	Andrade et al. [12]
Calcium	149.75	171.07	219.34	238.25
Copper	4.86	1.43	2.67	2.70
Iron	8.83	8.75	6.9	5.32
Phosphorus	899.33	832.9	995.66	1096.55
Magnesium	136.58	94.71	116.74	131.67
Manganese	4.40	3.35	2.77	1.66
Potassium	389.92	126.62	1025.49	1145.28
Sodium	101.44	435.06	381.97	417.44
Zinc	19.61	20.22	19.60	18.75

Notes: The last two columns are from the same author but refer to different diets.

**Table 5:** Vitamins of *Acheta domesticus* in four different breeding systems.

Vitamines (mg/100 g)	Payne et al. [67]	Ververis et al. [68]	Magara et al. [10]	Ververis et al. [68]
	Undried Matter		Dried Matter	
Vitamin A	0.0144	n.d.–0.023	<67.00 *	n.d.–0.040
β carotene	NA	<0.2–0.25	<0.02 *	NA
Vitamin B1	0.04	n.d.–1.21	0.04	0.24–1.66
Vitamin B2	3.41	n.d.–17.4	3.41	0.097–4.58
Vitamin B3	3.84	0.1–3.84	3.84	n.d.–4.51
Vitamin B5	2.3	2.03–2.63	2.30	4.30–4.42
Vitamin B6	NA	n.d.–0.23	0.23	NA
Vitamin B7	0.017	0.021	0.02	0.099–0.112
Vitamin B9	150	0.01–0.15	0.15	0.142–0.199
Vitamin B12	0.005	0.001–2.04	0.01	n.d.–0.009
Vitamin C	3.00	1.8–9.2	3.00	23.9
Vitamin D	0.64	NA	<17.15 *	NA
Vitamin E	2.26	1.15–15.13	1.32	3.68–332.06
Vitamin K	NA	7.84	NA	NA
Choline	NA	NA	151.90	NA

Notes: NA, not available/not reported in the source. n.d., not detected. * Values reported in secondary compilations may reflect unit inconsistencies for vitamins A and D (mg vs. µg) and/or reporting as detection limits; these entries are presented here in µg/100 g for comparability and should be read with care [10].

**Table 6:** Key techno-functional, physicochemical, and microbiological parameters reported for house cricket powder (values are indicative; methods vary across studies).

Parameter (Unit)	Typical Values and Interpretation	References
**Techno-functional properties**		
Water absorption capacity (WAC; %, *w*/*w*)	≈175% (≈1.75 g water/g powder). High WAC supports moisture retention and texture in bakery/snack systems.	[20,21]
Oil absorption capacity (OAC; %, *w*/*w*)	≈140% (≈1.40 g oil/g powder). High OAC can enhance mouthfeel and flavor retention in meat analogs and baked goods.	[20,21]
Emulsifying capacity (EC; %)	≈77.7%. Indicates ability to stabilize oil–water emulsions; relevant for meat emulsions, sauces and spreads.	[20,21,81]
Foaming capacity (FC; %)	≈15.9%. Reflects air incorporation; typically, lower than egg/plant proteins but can contribute in baked systems.	[20,21]
Foam stability (FS; %)	≈80.5%. Higher stability supports structure in aerated products when foams form.	[20,21]
Protein solubility (%, *w*/*w*)	≈30.5% under reported conditions; strongly pH- and salt-dependent, with lower solubility near pH ≈4 (isoelectric region) and higher solubility at alkaline pH. Solubility underpins emulsifying/foaming performance.	[11,21,81]
**Physicochemical properties**		
pH	≈6.8–6.9. Near-neutral pH supports broad compatibility with many formulations.	[20,21]
Moisture (%, *w*/*w*)	<5%. Low moisture contributes to shelf stability but may require formulation adjustments for hydration.	[20,21]
Water activity (aw)	≈0.30–0.40. Low aw limits microbial growth and supports storage stability.	[20,21]
**Microbiological profile**		
Pathogens (qualitative)	Typically, not detected when hygienic rearing and validated killing/drying are applied (e.g., *Salmonella* spp., *L. monocytogenes*).	[24,82]
Total viable counts (TVC; CFU/g)	Low counts reported for properly processed powders and stability during storage have been observed; reported values depend strongly on processing and storage.	[24,82]

**Table 7:** Selected food applications of *Acheta domesticus* powder and their main formulation outcomes.

Food Product	% Incorporation of Cricket Powder	Main Effects Observed	References
Meat emulsion (sausage)	5% and 10% replacement of lean meat and/or fat portions	Protein solubility was affected by pH and NaCl concentration. Water absorption capacity, emulsifying capacity and gel-forming ability were evaluated. Replacement up to 10% fortified protein and selected minerals without negative effects on cooking yield and instrumental texture.	[15]
Wheat flour for bread and fresh pasta	5%, 10% and 20%	Cricket powder affected functional and rheological properties of wheat-based formulations. Bread enriched with 10% cricket powder showed similar volume and crumb hardness to the control, while fresh pasta showed the best cooking behavior at 5% replacement.	[13]
Muffins	2%, 5% and 10%	Cricket powder increased protein content and decreased carbohydrate content. Muffins became darker and showed reduced hardness, springiness, chewiness and resilience, with no relevant change in cohesiveness. The 2% formulation was closest to the control in consumer acceptance.	[14]
Bread and biscuits	10%	Enrichment with cricket powder increased protein, iron and phosphorus content and reduced carbohydrate content. Bread and cookies became darker, while consumer acceptability and physical quality remained comparable to the control.	[16]
Burgers/beef patty	5%, 7.5% and 10% replacement of lean meat	Cricket powder increased protein content and reduced cooking loss and diameter reduction. Patties became firmer and browner. The 5% formulation showed the best balance between technological, sensory and cooking properties.	[17]
Shortbread cookies	10% and 20% replacement of wheat powder	House cricket powder increased protein content and improved nutritional value. The incorporation affected physical and sensory characteristics, with sensory scores tending to decrease as the level of insect powder increased.	[18]
Gluten-free bars	5%, 10%, 15%, 20% and 25%	Cricket powder increased total polyphenol content and antioxidant activity and reduced moisture content. Sensory evaluation was generally positive, although the highest level, 25%, showed the lowest scores for taste and overall pleasantness.	[19]
Chocolate chip cookies	5%, 7.5% and 10%	Cricket powder increased protein content and modified functional, rheological and sensory properties. Cookies became darker and softer. The 5% formulation had the best sensory performance, while 10% showed the lowest liking scores.	[24]

Note: Incorporation levels are reported as % (*w*/*w*) of cricket flour/powder in the formulation; ranges indicate the minimum–maximum levels tested in the cited study.

## Data Availability

No new data were created or analyzed in this study. Data sharing is not applicable to this article.
